# Amerindian ancestry proportion as a risk factor for inflammatory bowel diseases: results from a Latin American Andean cohort

**DOI:** 10.3389/fmed.2023.1258395

**Published:** 2023-10-27

**Authors:** Tamara Pérez-Jeldres, Fabien Magne, Gabriel Ascui, Danilo Alvares, Matias Orellana, Manuel Alvarez-Lobos, Cristian Hernandez-Rocha, Lorena Azocar, Nataly Aguilar, Alberto Espino, Ricardo Estela, Sergio Escobar, Alejandra Zazueta, Pablo Baez, Verónica Silva, Andres De La Vega, Elizabeth Arriagada, Carolina Pavez-Ovalle, Alejandro Díaz-Asencio, Dante Travisany, Juan Francisco Miquel, Eduardo J. Villablanca, Mitchell Kronenberg, María Leonor Bustamante

**Affiliations:** ^1^Department of Gastroenterology, School of Medicine, Pontificia Universidad Católica de Chile, Santiago, Chile; ^2^Department of Gastroenterology, Hospital San Borja Arriarán, Santiago, Chile; ^3^Department of Microbiology, Faculty of Medicine, Institute of Biomedical Sciences (ICBM), Universidad de Chile, Santiago, Chile; ^4^La Jolla Institute for Immunology, San Diego, CA, United States; ^5^MRC Biostatistics Unit, University of Cambridge, Cambridge, United Kingdom; ^6^Department of Computer Science, Faculty of Physical Sciences and Mathematics, Universidad de Chile, Santiago, Chile; ^7^Center of Medical Informatics and Telemedicine, University of Chile, Santiago, Chile; ^8^Núcleo de Investigación en Data Science, Facultad de Ingeniería y Negocios, Universidad de las Américas, Santiago, Chile; ^9^Division of Immunology and Allergy, Department of Medicine, Solna, Karolinska Institutet and University Hospital, Stockholm, Sweden; ^10^Department of Molecular Biology, University of California San Diego, La Jolla, CA, United States; ^11^Department of Human Genetic, Faculty of Medicine, Institute of Biomedical Sciences (ICBM), Universidad de Chile, Santiago, Chile; ^12^Fundación Diagnosis, Santiago, Chile

**Keywords:** inflammatory bowel disease, single nucleotide polymorphism (SNP), ancestry, Latin American, genetics

## Abstract

**Background and aims:**

Latin American populations remain underrepresented in genetic studies of inflammatory bowel diseases (IBDs). Most genetic association studies of IBD rely on Caucasian, African, and Asian individuals. These associations have yet to be evaluated in detail in the Andean region of South America. We explored the contribution of IBD-reported genetic risk variants to a Chilean cohort and the ancestry contribution to IBD in this cohort.

**Methods:**

A total of 192 Chilean IBD patients were genotyped using Illumina's Global Screening Array. Genotype data were combined with similar information from 3,147 Chilean controls. The proportions of Aymara, African, European, and Mapuche ancestries were estimated using the software ADMIXTURE. We calculated the odds ratios (ORs) and 95% confidence intervals (CIs) for gender, age, and ancestry proportions. We also explored associations with previously reported IBD-risk variants independently and in conjunction with genetic ancestry.

**Results:**

The first and third quartiles of the proportion of Mapuche ancestry in IBD patients were 24.7 and 34.2%, respectively, and the corresponding OR was 2.30 (95%CI 1.52–3.48) for the lowest vs. the highest group. Only one variant (rs7210086) of the 180 reported IBD-risk SNPs was associated with IBD risk in the Chilean cohort (adjusted *P* = 0.01). This variant is related to myeloid cells.

**Conclusion:**

The type and proportion of Native American ancestry in Chileans seem to be associated with IBD risk. Variants associated with IBD risk in this Andean region were related to myeloid cells and the innate immune response.

## Introduction

Inflammatory bowel disease (IBD), including Crohn's disease (CD) and ulcerative colitis (UC), is a complex and heterogeneous disease driven by the confluence of multiple environmental and genetic variables that alter the immune–microbiome axis (1).

Although it was previously considered a disorder that affects individuals of European ancestry, IBD has emerged as a global disease (2). Moreover, IBD epidemiology is changing with an increased incidence in previously low-incidence areas, such as Latin America and Africa (3, 4). The current rise in IBD incidence is parallel to industrialization, westernization, and urbanization, which might reflect the influence of changes in environmental exposure to IBD development (3). In fact, early-life events such as childbirth mode, breastfeeding, antibiotics exposure, and later childhood events have been considered potential IBD risk factors (5). Despite this knowledge, the mechanisms through which environmental factors may be causally related to IBD are not well-elucidated. Impairment in immune tolerance, increased stress levels, westernized diet, lifestyle, and exposure to pollutants may be some potential contributors (3, 6).

On the other hand, it has been established that genetic factors play a role in IBD risk, and SNP heritability has been estimated to be 25% for overall IBD, 27% for UC, and 21% for CD (7). The results from genome-wide association studies (GWAS) point to an association with over 240 distinct single-nucleotide polymorphisms (SNPs) that explain a sizeable fraction of the genetic variability in the occurrence of IBD (8, 9).

Despite the increasing recognition of the IBD global relevance, genetic research on IBD has focused on Europeans, and most IBD susceptibility loci have been identified in this group (10). Even the non-European studies are mainly limited to the East and South Asian populations, including Korea, Japan, and north India, contributing a finite number of loci due to their small sample sizes (11–13).

The increase in incidence and prevalence of IBD has also been observed in the Chilean population classified as Latin Americans (14). Currently, no registered epidemiological information is available on the prevalence of IBD in Chile. However, the reported prevalence rate in Brazil is 38.2 per 100,000 persons (15, 16).

Interestingly, Latin Americans are highly heterogeneous regarding Native American ancestry, with differences in the admixture proportions of European, African, and Amerindian between and within countries (17, 18).

Currently, there is a lack of knowledge of the contribution of ancestry to IBD in the Andean region of the South American population. Ongoing genetic studies are focused on Hispanics, both foreign-born and US-born Hispanic-Americans living in the US, and Puerto Rican IBD patients (19).

We conducted our study specifically on a population from Chile, which is the result of genetic admixture between Native Americans, Spaniards who arrived in Chile mostly in the mid-sixteenth century, enslaved Africans who reached Chile in the seventeenth century, and migrations from Europeans in the nineteenth and twentieth centuries (20, 21). Furthermore, two large groups are present in indigenous (aboriginal) people (Mapuche and Aymara), and the Amerindian ancestry proportion is higher in the north and the south of Chile. In contrast, European ancestry is highest in the central area (22, 23). On average, Chileans are 53% European and 42% Amerindian (disaggregated into 18% Aymara and 25% Mapuche) (22).

This study aimed to explore the contribution of genetic ancestry to IBD risk in Chileans and to test for previously reported IBD-risk variants, both independently and in conjunction with genetic ancestry. We estimated European, African, Mapuche, and Aymara ancestry proportions. Then, we calculated the odds ratios (ORs) and 95% confidence intervals (CIs) according to ancestry proportion. We used a case–control univariate and multiple regression analysis for the additive model and three genotypes to test associations between IBD and susceptibility-reported risk variants.

## Methods

### Recruitment of IBD patients

The design is a prospective, observational study. Chilean patients attending Hospital San Borja Arriarán (HSBA) with IBD diagnoses for at least three months were invited to participate. The IBD diagnosis was supported by clinical, endoscopic, histologic, and imaging findings according to the International Disease Classification Criteria (24–27). Ethics approval was obtained from the Institutional Review Boards of the Pontificia Universidad Católica de Chile (IRB:220228001) and the Servicio de Salud Metropolitano Central/HSBA (IRB:43/2022). All individuals provided written informed consent. IBD standard clinical information and medical history were collected.

The HSBA, placed in Santiago, the central zone of Chile, belongs to the Chilean Public Health System, has 56 medical specialties, 549 beds (available for the public health network), and an IBD center care for more than 500 IBD patients. The patients have similar socioeconomic levels, according to the scale of the Association of Market Researchers and Public Opinion, Chile (lower middle class and working class, C3 and D, respectively) (28, 29).

### Genotyping

In total, 5 ml of blood was collected from each participant and stored in plastic vacutainer tubes containing ethylenediaminetetraacetic (EDTA). DNA from peripheral blood was extracted using an Invisorb Blood Universal Purification Kit (Invitek, # ref 1031150200), following the manufacturer's instructions. Samples were stored at −80°C until genotyped at Erasmus MC-Netherlands using Illumina's Infinium Global Screening Array, and 725.497 SNPs were investigated.

### Genotyping QC

Genetic variants were filtered to exclude non-autosomal polymorphisms, variants with a missing call rate of 5%, and variants with a minor allele frequency of <5%. Linkage-disequilibrium (LD) pruning was performed for variants at r^2^ > 0.1.

The sample QC process for cases and controls involves multiple steps. First, samples with relatedness or duplication were excluded. The identity by descent (IBD) measurement was used to assess relatedness. Samples with an IBD value close to 1 were considered duplicates and removed from the analysis. Individuals with an IBD value exceeding 0.185 were regarded as first or second degree related and thus excluded from the analysis. The X chromosome homozygosity rate was estimated to detect sex discrepancies. Men were expected to have a homozygosity rate above 0.8, while women were expected to have a rate below 0.2.

The sample call rate was determined by calculating the proportion of missingness across SNPs. It measures the amount of missing data by counting missing genotype calls and dividing it by the total possible calls. In this study, variants with missing call rates above 5% were excluded, considering the sample size and the focus on variants with a significant effect or relevance to the phenotype.

LD pruning was performed using an r^2^ > 0.1 to avoid redundancy, improve computational efficiency, and select independent markers.

### Control samples

Genotypic information from 3,147 individuals of Chilean descent was included in the study. Controls were genotyped using Illumina's Human 610-Quad BeadChip. Genotypes, as well as sociodemographic information, were obtained as part of a case–control study, designed to investigate causative factors of gallbladder cancer in Chileans. Recruitment criteria, as well as ethical certifications obtained for the original study, which were led by Dr. Justo Lorenzo-Bermejo, have been previously described (30).

In brief, all control individuals provided written informed consent under the supervision of all the Chilean institutions involved. Neither IBD nor other immune-mediated illnesses were explored while collecting this sample. The proportions of Chilean controls affected by these should be representative of the corresponding proportions in the general population that gave rise to the cases. Thus, only a small number of individuals could be expected to be affected among the control sample. Based on their income and occupation, all individuals belonged to the C2, C3, D, or E socioeconomic strata (lower middle and lower classes) (28, 29). Although the control sample was recruited from all regions of Chile, their socioeconomic status and demographic characteristics are similar to those of the cases. According to previous reports, their ancestry components (see Results) represent the whole country and are similar to those from health centers in Santiago (22). To comply with data-agreement policies from the original study, the authors of the present study did not receive access to individualized data; aggregated data and analysis results were provided by the principal investigator of the original study as part of an academic collaboration (see Acknowledgment Section).

All samples were genotyped using SNP arrays of a similar coverage as the one used for patients (approximately 700,000 SNPs).

### Estimation of genetic ancestry

Ancestry was estimated using ADMIXTURE software (31) for supervised estimation of individual European, African, Mapuche, and Aymara ancestries. Because the authors of the present study did not have access to individualized data from control subjects, ancestry estimates were carried out by the collaborating researchers (see Acknowledgment section).

Reference individuals were used for inferring the individual ancestry ratios of Chileans. European ancestry surrogates were 99 Utah residents of Northern and Western European ancestry (CEU) and 107 individuals from the Iberian population in Spain (IBS). African ancestry surrogates were 108 Yorubans in Ibadan, Nigeria (YRI). The CEU, IBS, and YRI populations belong to the 1,000 Genomes Project (32). The most numerous indigenous people in Chile are Mapuche in the south and Aymara in the north, represented in this study by 28 and 63 reference subjects, respectively (33–35). These individual ancestry references correspond to the same subjects used to estimate ancestry controls. To determine the optimal number of ancestral populations or clusters for a supervised analysis with a known reference ancestry panel, an embedded cross-validation (CV) algorithm was employed to identify the number of ancestral populations that yielded the lowest cross-validation error (K). Both 5-fold and 10-fold cross-validation approaches were utilized. The cross-validation error for each *K*-value was calculated and plotted, with K represented on the x-axis and the cross-validation error represented on the y-axis. The lower cross-validation error was 0.55438 for cases and 0.55973 for controls for K = 4.

We conducted a genetic principal component analysis using the EIGENSTRAT function to analyze the population structure, and all the data ran simultaneously (36).

### Statistical methods

Using the statistical software R version 4.2.1, we calculated the odds ratios (ORs) and 95% confidence intervals (CIs) for gender, as well as age, and ancestry proportions grouped into quartiles using the library “Epitools.”

We investigated 200 SNPs previously associated with IBD (83 of them had pGWAS value < 5 × 10^−8^) (8, 9) in our Chilean IBD group (Supplementary material 1). Associations between identified IBD susceptibility variants were tested using a case–control univariate and multiple regression analysis for the additive model. Since cases and controls had not been matched by age, sex, and ancestry proportion, covariates such as age, sex, and ancestry were included to account for stratification and avoid confounding effects from demographic factors in multiple regression models. Then, we estimated the OR and 95% CI for IBD in the Chilean Cohort. In addition, we also tested the three-genotype model.

The same models were repeated to evaluate the association between IBD-risk SNPs and the proportion of Mapuche (PMA). We stratified the ancestry ratio into two categories (low vs. high) based on the quartile 1 (24.7%) of the ancestry ratios.

A *chi*-square association test was performed to calculate *p*-values for each model and SNP, and Bonferroni correction was used to calculate the adjusted *p*-values. For the analysis mentioned above, we used R version 4.2.1 and the libraries “readxl,” “rapportools,” and “dplyr”.

## Results

A total of 192 IBD Chilean patients were genotyped using Illumina's Infinium Global Screening Array, and data from 186 were used for ancestry analysis. In this group, 75% of the patients had diagnoses of UC, whereas 25% had CD. The median age was 49 years (17–81), with a median age at diagnosis of 36 years (7–73), and the median of the duration of disease was 8 years (range 1–47 years). In this IBD patient cohort, 36% had extra intestinal manifestations, more than 50% had a history of hospitalization caused by IBD, and only 15% had a history of bowel resection surgery. More than 50% of the IBD patients were under treatment with thiopurines, and only 17% were under anti-TNF therapy. According to the Montreal Classification, 35% of UC had extensive colitis, 31% had left colitis, 25% had proctitis, and in 9%, this information was not available. In the CD group, only 4% had a diagnosis before the age of 17 years. The colonic extension (L2) was most frequent (46%), followed by the ileocolonic (L3) extension. Only 5% of CD cases had an upper digestive tract compromise (L4), and 54% had perianal involvement. The most frequent CD phenotype was structuring (B2 = 37%), followed by 33% inflammatory (B1). The complete clinical characterization of the investigated IBD patients is presented in Table 1.

**Table 1:** Clinical data of the Chilean IBD patients.

**Clinical variable**	***n =* 184**
Ulcerative Colitis/Crohn Disease	138 (75%)/46 (25%)
Age, years, median (max–min)	49 (17-81)
Age, years, at diagnoses (median, min-max)	36 (7–73)
Disease Duration, years, (median, min-max)	8 (1–47)
Extraintestinal manifestation (no/yes)	116 (63%)/68 (37%)
Hospitalization (no/yes/no data)	83 (45%)/99 (53.8%)/2 (0.2%)
Smoking (no/yes/no data)	153 (83.2%)/30 (16.3%)/1 (0.5%)
**Hemoglobin (g/L)**	
Normal range 12–18, mean (max–min)	13.84 (8.80–17.60)
**White cell count** × **10**^6^**/L**	
Normal range 4000–11000, mean (max–min)	7138 (2690–16740)
**Platelets**, × **10**^6^**/L**	
Normal range 150000–450000, mean (max–min)	294452 (28000–602000)
**C reactive protein (mg/dL)**	
Normal range < 0.5, mean (max–min)	2.55 (0.03–30)
**Albumin (g/L)**	
Normal range (3.5–5.5), mean (max–min)	4.40 (2.9–5.2)
Resective surgery (no/yes)	156 (85%)/28 (15%)
**History infection for clostridiodes difficile**	
(No/yes/no data)	162 (88%)/20 (11%)/2 (1%)
Current use of steroids (no/yes)	151 (82%)/33 (18%)
Anti-TNFa use (no/yes)	152 (83%)/32 (17%)
Thiopurines/Inmunosupresor use	116 (63%)/68 (37%)
Naïve anti TNFa (no/yes)	38 (21%)/146 (79%)
**Montreal ulcerative colitis (*****N*** = **138)**	
Extensive colitis (E3)	48 (35%)
Left colitis (E2)	43 (31%)
Proctitis (E1)	34 (24.6%)
No registered	13 (9.4%)
**Montreal Crohn's disease (*****n** =* **46)**	
**Age**	
A1/A2/A3/No registered	2 (4%)/21 (46%)/22 (48%)/1 (2%)
**Localization**	
Ileal (L1)	6 (13%)
Colonic (L2)	21 (45.7%)
Ileocolonic (L3)	16 (34.8%
No data	3 (6.5%)
Upper compromise (L4) (no/yes/no registered)	40 (87%)/ 5 (11%)/1 (2%)
**Behavior**	
B1 (inflammatory)	15 (33%)
B2 (structuring)	11 (24%)
B3 (penetrating)	17 (37%)
No registered	3 (6%)
Perianal disease (yes/no)	25 (54%)/21 (46%)

Anti-Tumor Necrosis Factor alpha = Anti-TNFa; A1, age at diagnoses Crohn's disease less than < 16 years; A2, age at diagnoses Crohn's disease 17–40 years; A3, age at diagnoses Crohn's Disease more than >40 years.

Table 2 shows the demographic and genetic ancestry characteristics of the study population and their associations with IBD risk. As shown in Table 1, our findings suggest that Chilean individuals aged 25–35 years old and those older than 50 years may have a higher risk of developing IBD with OR = 1.74, CI = 1.14–2.63, and OR = 1.87, CI = 1.23–2.84 (*p* = 6 × 10–6), respectively. These results agree with the reported in the literature since IBD has two incidence peaks: the first occurs between the ages of 20 and 39 years, while the second is observed between the ages of 50 and 70 years (37). In our study group, men had a higher IBD risk, but the sample size of men in the cases group was smaller than the number of women.

**Table 2:** Demographic and genetic ancestry data.

**Variable**	**Category**	**Cases (*n =* 186)**	**Controls (*n =* 3,147)**	**OR**	**95%CI**	**PVal**
Age (years)	< 25	49	626	Reference		6 × 10^−6^
	25–35	45	999	1.74	1.14–2.63	
	36–49	47	443	0.74	0.48–1.12	
	50+	45	1,079	1.87	1.23–2.84	
Sex	Female	117	1,603	Reference		0.002
	Male	69	1,544	1.63	1.20–2.21	
Mapuche ancestry	< 24.7%	47	627	Reference		5 × 10^−6^
	24.7–30.2%	46	485	0.79	0.51–1.20	
	30.2–34.2%	46	592	0.96	0.63–1.40	
	34.2–87.3%	47	1,443	2.3	1.52–3.48	
Aymara ancestry	< 8.2%	47	1,244	Reference		1.5 × 10^−14^
	8.2–10.6%	46	443	0.36	0.23–0.55	
	10.6–12.7%	46	303	0.24	0.16–0.38	
	12.7–26.3%	47	1,157	0.94	0.62–1.44	
European ancestry	< 52.1%	47	1,667	Reference		5 × 10^−16^
	52.1–56.6%	46	651	0.39	0.26–0.60	
	56.6–62.5%	46	522	0.31	0.21–0.48	
	62.5–99.1%	47	307	0.18	0.12–0.28	
African ancestry	< 1.3%	47	848	Reference		0.09
	1.3–1.9%	46	612	0.73	0.48–1.12	
	1.9–2.8%	46	668	0.8	0.52–1.22	
	2.8–20.0%	47	1019	1.2	0.79–1.82	

OR, odds ratio; CI, confidence interval; PVal, p-value.

The first and third quartiles of PMA in IBD patients were 24.7 and 34.2%, respectively, and the corresponding OR was 2.30 (95% CI 1.52–3.48) for the lowest PMA vs. the highest group. Significant differences in risk were also observed for Aymara and European ancestries. In our study, the first and third quartiles of Proportion of European Ancestry (PEA) were 52.1 and 62.5%, respectively, and the corresponding OR was 0.18 (95%, CI 0.12–0.28) for the highest PEA vs. the lower group. Thus, a higher proportion of European ancestry was associated with lower IBD risk (Table 2).

**Figure 1:**
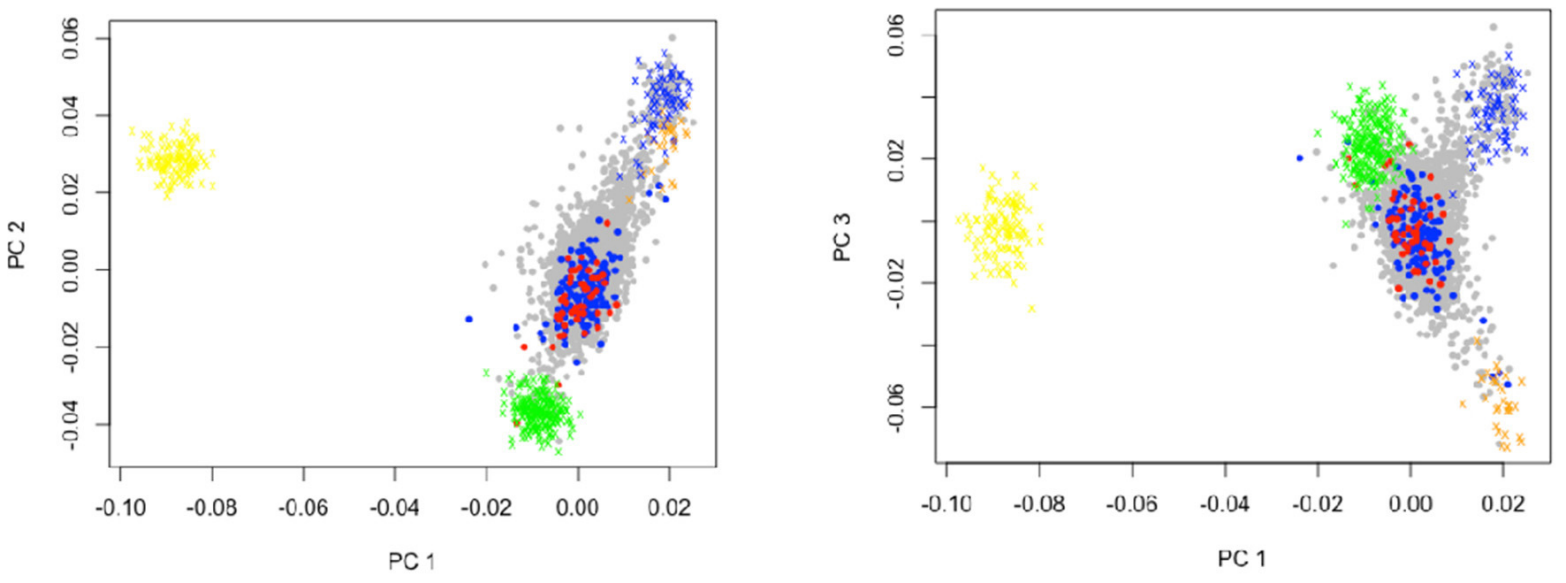
Genetic principal component analyses of study individuals and Mapuche, European, African, and Aymara reference individuals. This figure shows the distribution of ancestry informative markers (ATMs) in the sample. PC1 distinguished Native American and European from Africans. The PC2 separated Native American from European ancestry components. CD and UC patients revealed greater European and Mapuche ancestry influences in our study population. The PC3 distinguished Mapuche from European and Aymara. Crosses represent reference populations, orange = Mapuche, blue = Aymara, green = European, yellow = African. Dark blue dots represent Ulcerative Colitis patients, and red dots Crohn's disease patients. PC = Principal Component.

In Figure 1, the genetic principal component analysis (PCA) shows the distribution of ancestry informative markers (AIMs) in the sample, affected individuals indicated in red for CD cases and blue for UC cases. The first principal component (PC1) distinguished Native American (light blue crosses for Aymara, and orange crosses for Mapuche) and European (green crosses) from African (yellow crosses) ancestry. The second principal component (PC2) separated Native American from European ancestry components. CD (red dots) and UC (dark blue dots) patients revealed greater European and Mapuche ancestry influences in our study population. The third principal component (PC3) distinguished Mapuche from European and Aymara ancestries. Interestingly in PC3, the Aymara group is closer to European than Mapuche. In the IBD group, the average Mapuche proportion was 30%, Aymara proportion was 11%, European proportion was 57%, and African proportion was 2%, whereas in the control group, the average proportions were 34, 9, 52, and 2%, respectively (30).

**Figure 2:**
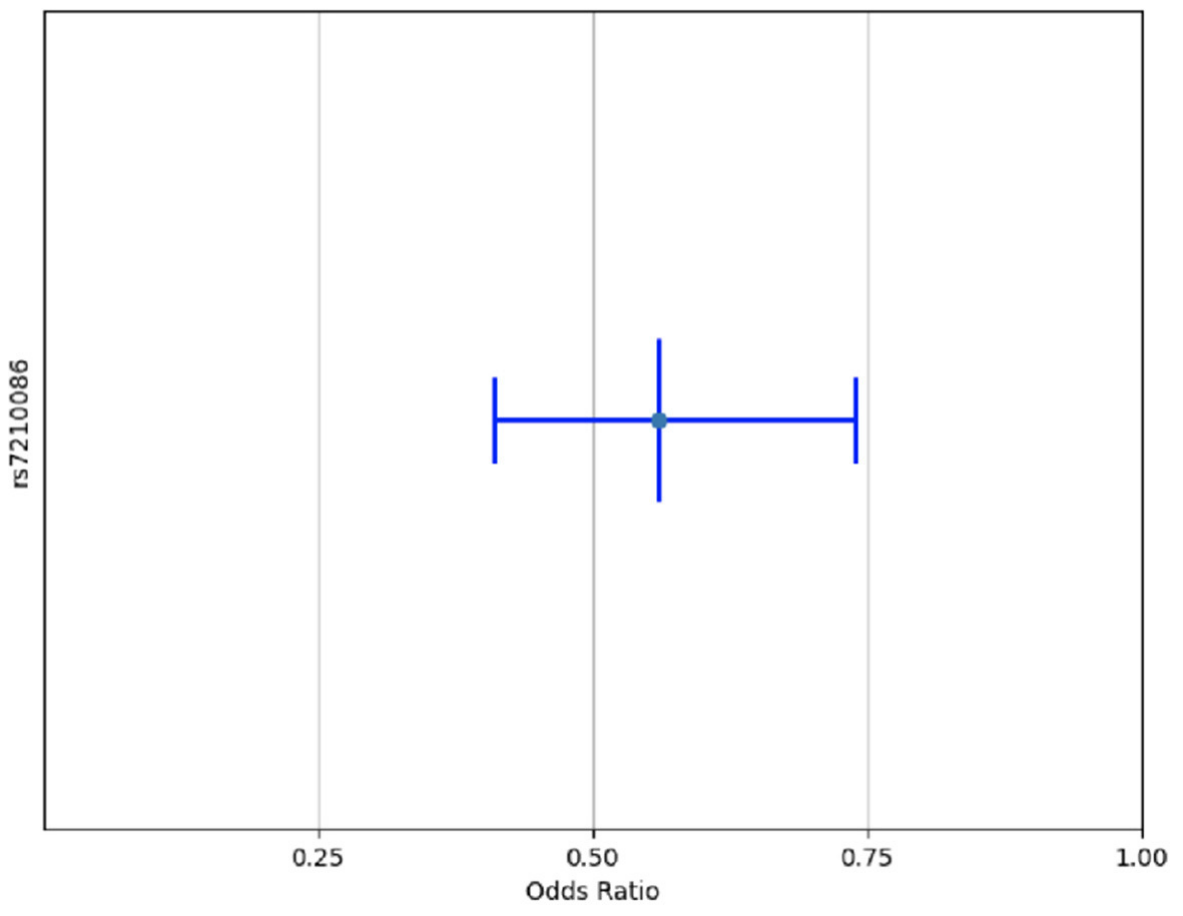
Variant rs7210086 associated with risk of IBD in Chilean Population. Only one variant of the 180 reported IBD-risk related SNPs was associated with IBD in Chilean cohort multivariant multiple regression models. A x^2^ association test was performed to calculate *p*-values for each SNP and adjusted by *post-hoc* Bonferroni correction. Only SNPs with an adjusted *p* < 0.05 were plotted.

From the initial selection of 200 candidate SNPs (Supplementary material 1), 180 ibd-risk SNPs were available for case–control regression analysis for the additive model. Only rs7210086-A (OR = 0.56, CI 0.41–0.74, adjusted *P* = 0.01) was associated significantly after adjusting *p*-value in the multivariate analysis (Table 3, Figure 2), and the same SNP was associated with IBD in three-genotype model (Supplementary Table 1). When stratifying by ancestry, considering a PMA higher than 24.1%, we do not find a significant association with IBD, as shown in Table 4 (Supplementary materials 2–4). Table 5A describes the representative frequency of the rs7210086 in the Chilean Cohort compared with others. The frequency allele of rs7219986-A was 0.839 for Chilean IBD, 0.746 for Chilean controls, and 0.832 in the global population (Ensembl genome data set), respectively. The comparison of the rs7219986 genotypes of Chilean control with other populations was also significantly different (Table 5B).

**Table 3:** Single nucleotide polymorphisms associated with IBD in the studied group: additive models.

**Model additive**						
**Type model**	**SNP**	**OR**	**2.5%**	**97.5%**	**PVal**	**Adjusted** ***P*****-value**
Univariate	rs7210086	0.56	0.42	0.74	0.00006	0.01
Multivariate		0.56	0.41	0.74	0.00007	0.01

SNP, single nucleotide polymorphism; OR, odds ratio; CI, confidence interval; PVal, p-value.

**Table 4:** Influence of proportion Mapuche genetic ancestry on the association of rs7210086 SNPs and IBD.

**Additive high Mapuche** ^*^						
**Type model**	**SNP**	**OR**	**2.5%**	**97.5%**	**Pval**	**Adjusted** ***P*****-value**
Univariate	rs7210086	0.58	0.41	0.79	0.001	0.38
Multivariate		0.55	0.39	0.76	0.0004	0.17

^*^High Mapuche corresponds with proportion of Mapuche ancestry higher than 24.7% as shown in Table 1. SNP, single nucleotide polymorphism; OR, odds ratio; CI, confidence interval; PVal, p-value.

**Table 5A:** Characterization of rs7210086 variant in the Chilean cohort.

**SNP**	**Genotypes**	**Cases *n =* 186**	**Controls *n =* 3,147**	**Risk allele**	**Gene name**	**Description**	**Location**
rs7210086	AA	132	1,745	A	LINC00511, SLC39A11	Intergenic variant	17:72645559
	AC	48	1,206				
	CC	6	196				

**Table 5B:** Comparison of allele and genotype frequencies for rs7210086 among Chileans and other populations reported in Ensembl genome data set.

**SNP**	**Group**	**Allele**	**Genotype**	***P*-value (Chi-square test)**
rs7210086	All	A = 0.832	AA = 0.695 (1740)	< 2.2 × 10^−6^
		C = 0.168	AC = 0.275 (688)	
			CC = 0.030 (76)	
	European	A = 0.833	AA = 0.688 (346)	1.47 × 10^−8^
		C = 0.167	AC = 0.290 (146)	
			CC = 0.022 (11)	
	Latin American^*^	A = 0.716	AA = 0.565 (196)	0.367
		C = 0.239	AC = 0.392 (136)	
			CC = 0.043 (15)	
	Controls (Chile)	A = 0.746	AA = 0.555 (1745)	Reference
		C = 0.254	AC = 0.383 (1206)	
			CC = 0.062 (196)	
	IBD (Chile)	A = 0.839	AA = 132 (0.710)	0.0002
		C = 0.161	AC = 48 (0.258)	
			CC = 6 (0.032)	

SNP, single nucleotide polymorphism. ^*^Colombian, Peruvian, Mexican, and Puerto Rican.

**Figure 3:**
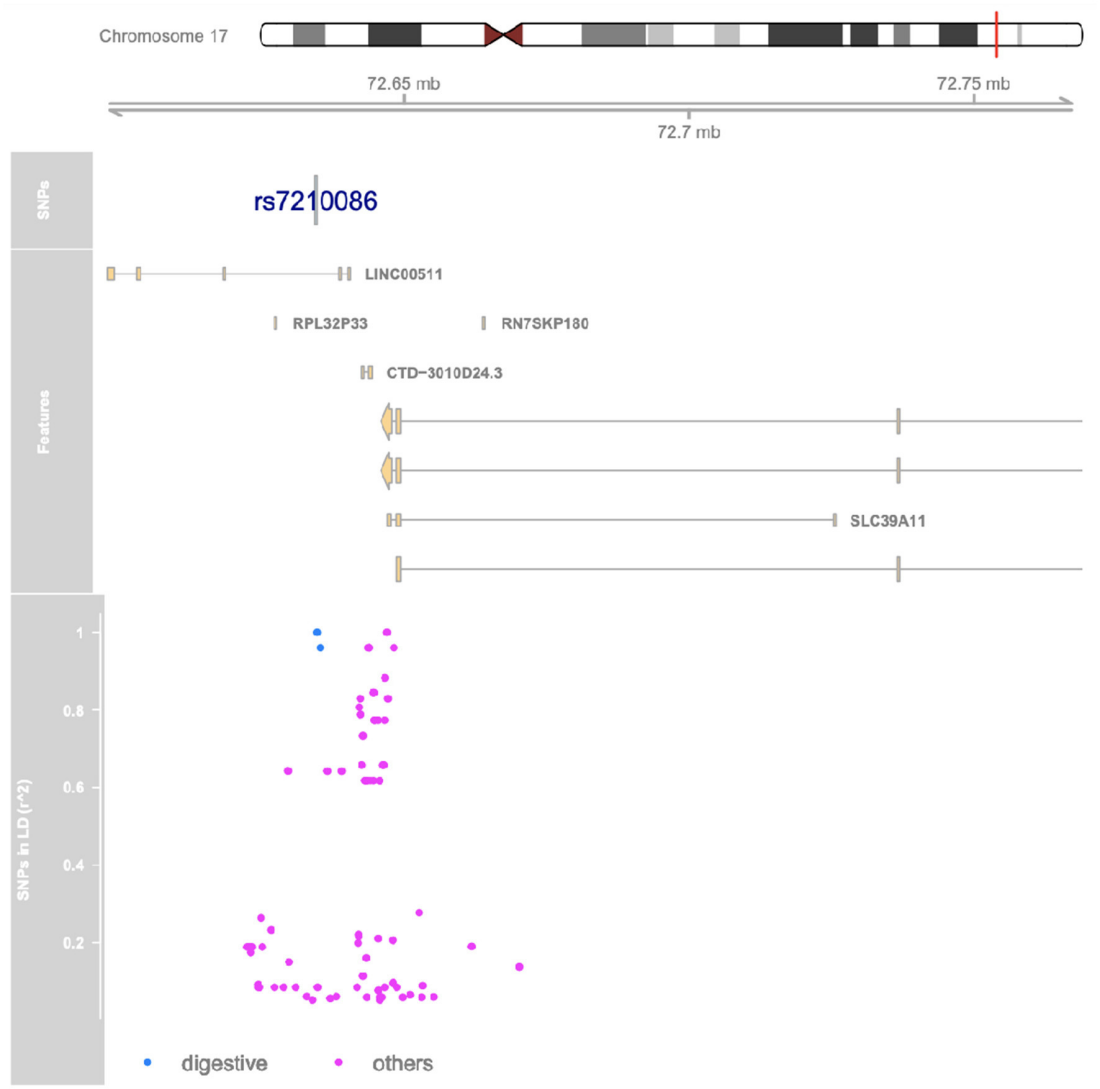
Variants in linkage disequilibrium with SNPs significantly associated with IBD in Chilean patients. The red line marks the region of interest on chromosome. Genetic context of rs7210086 on chromosome 17 of hg19. Genes found in this region are plotted in yellow. Variants in linkage disequilibrium with rs7210086 are plotted below indicating r2 values with rs7210086 for each one. Variants that have been previously associated with phenotypical traits by GWAS and are indicated in either blue for digestive disorders, or violet for others. The rs17780256 is represented by blue dots.

We performed linkage disequilibrium (LD) analyses for rs7210086 because associations between genetic variants and traits are usually in non-coding regions with strong LD, where a single causal variant is assumed to underlie the association. Among the variants in LD with rs7210086, there is rs17780256, which previously has been related to chronic inflammatory diseases such as IBD, among others. Supplementary material 5 displays the variants in LD with rs7210086 and their location, consequences, r2, and phenotypes. The marker rs7210086 is located on chromosome 17, as shown in Figure 3. The variants in linkage disequilibrium with rs7210086 are plotted and associated with phenotypic traits by GWAS, such as digestive disorders or others.

## Discussion

Our data explore the risk of known genetic variants associated with IBD in the Chilean population, a previously unstudied population from the Andean Region. Most studies are focused on Mexicans, Puerto Ricans, and Latinos living in the US, with few reports analyzing SNPs related to IBD in South American populations (19, 38–40). The present study is the first to report the contribution of genetic ancestry and IBD risk in a South American cohort, analyzing a population characterized by a unique ancestry admixture. Latin Americans have been considered homogenous in most GWAS, ignoring the difference in admixture variation between Latin American countries. For example, Puerto Ricans and Colombians have higher percentages of African ancestry than Chileans and Mexicans, potentially translating into differential disease risk, according to the proportion of ancestries (33). Furthermore, Latin Americans are highly heterogeneous regarding their Native American Ancestry. For example, Aymara and Mapuche are common for Chileans and Aztec and Maya for Mexicans (33).

In our study, a higher PMA was related to higher IBD risk; on the other hand, after stratifying for PMA, we only found 1 out of 180 known risk SNPs to be significantly associated with disease. *Post-hoc* power calculation shows that our study has a power of more than 90% for variants with a relative risk of 1.7 or more and 60% for variants with a relative risk of 1.3 (41).

From the previously reported risk variants, in this study, we found only the rs7210086-A, OR 0.56 (CI 0.41–0.74, *P*-value adjusted multivariate analysis < 0.01). This result suggests that genetic risk factors may be specific to the Chilean population, including both the population results from admixture between Spaniards and Native Americans, as well as pure Native Americans such as the Mapuche, have thus not been captured by previous studies, but this requires increasing sample size to evaluate adequately. This underscores the relevance of expanding the available genetic information since an important limitation of the first and second waves of genetic studies in IBD is that they were carried out in white populations of European ancestry (42). Subsequently, studies including Iranian, Asian, Indian, and African American descent revealed broad similarities with that observed in the initial GWAS but with ethnicity-specific differences in direction and size effect (9, 42, 43). Known genetic factors predict a smaller IBD fraction in Hispanics and probably even a lower fraction in Latin Americans (42). GWAS are required in these populations, including Mapuche and others.

In our cohort, only the rs7210086 was associated with IBD risk. The A-allele has been related to IBD risk with an OR = 1.11 (1.06–1.16, pGWAS = 2 × 10^−9^) (8, 44). Interestingly, in our study, the alternative allele (C) conferred higher risk (adjusted *P* = 0.01). This variant falls in an intergenic area—the closest 5′ and 3′ genes are the *SCL39A11* and *LINC00511*, respectively (44). As mentioned above, we performed an LD analysis for this variant. LD is a phenomenon that describes SNPs located nearby on a chromosome segregating together more often than expected by chance. The findings from association studies and functional effects attributed to SNPs could be thus confounded by LD, with one or a few SNPs responsible for the functional effect, whereas the others could only serve as markers (45). Among the variants in LD with this variant is rs17780256 (Figure 3, blue dots, Supplementary material), which also maps to the *SLC39A11* gene. The rs17780256 has been related to chronic inflammatory diseases such as ankylosing spondylitis, CD, psoriasis, primary sclerosing cholangitis, and UC (46).

*SLC39A11* encodes a zinc transporter that plays a crucial role in Zn homeostasis, which is necessary for the innate immune system, especially for maintaining the function of macrophages (47). *SLC39A11* also regulates the expression of calprotectin in myeloid cells, controlling the expression levels of S100A8 and S100A9, the two subunits of calprotectin, an important IBD biomarker for monitoring disease activity (48). Calprotectin modulates the inflammatory response by stimulating leukocyte recruitment and inducing cytokine secretion (48).

The underlying mechanism by which *SLC39A11* is related to calprotectin levels is not known. The *SLC39A11* IBD gene has been shown to lead to increased expression of prostaglandin E2 receptor subtype 2 (*PTGER2*). (49). It is speculated that upon activation of PTGER2 by prostaglandin E2, adenyl cyclase is activated, which converts adenosine triphosphate (ATP) to cyclic adenosine monophosphate (cAMP). cAMP activates protein kinase A (PKA), which activates signal transducer and activator of transcription 3 (STAT3) by phosphorylation (49). STAT3 binds to the S100A8 and S100A9 promoters, inducing their expression. Since *SLC39A11* encodes a relatively poorly characterized metal ion transporter, which is believed to transport zinc ions, its role may be related to its control of zinc, which is required to stabilize S100A8 and S100A9 promoters, such as ZBTB40 and other zinc finger proteins. Then, ZBTB40 can act as a transcriptional modulator of S100A8 and S100A9, and its association with myeloid leukocyte activation, neutrophil activation, toll-like receptor binding, and immune activation has been described (49). In addition, *SLC39A11* expression throughout the intestinal mucosa regulated by zinc intake may implicate its role in these tissues, helping to maintain mucosal integrity and function (50, 51).

Our results encourage us to evaluate the impact of Mapuche ancestry on the immune response, which might translate into different phenotypes and therapy responses to IBD Chilean patients in comparison to Europeans. Individuals from different populations have differences in their susceptibility to chronic inflammatory disorders, infectious diseases, and autoimmune disorders. For systemic sclerosis, tuberculosis, systemic lupus erythematosus, septicemia, and psoriasis, Europeans and African Americans exhibit an up to 3-fold difference in prevalence (52).

Ethnic differences in the immune response show that descendants of Africans respond more strongly to infection than European descendants. Nédélec et al. (52) showed that differences in the transcriptional response to human infection are under strong genetic influence, determined by their ancestry and recent natural selection events.

Integration of population genetics with functional genomics in different populations might reveal the changes in immune functions related to the effect of natural selection on the evolution of the immune system and the history of past epidemics (53). Recently, Barreiro et al. proposed a plausible model for the prevalence of specific alleles involved in immune function. They demonstrated the effect of the rs2549794 variant of the *ERAP2* gene on macrophage function and response to *Yersinia pestis* infection. As *Yersinia pestis* caused one of the most significant pandemics recorded to date (Black Death), it is suspected that these allelic frequencies are strongly influenced by selection. Furthermore, variants of this gene have been linked to IBD (54). The authors conclude that retaining the alleles that became advantageous during the Black Death confers an increased risk of autoimmune disorders in the current population (54). Thus, the difference in epidemiological history in a population could explain differences in people from different geographies exposed to various environmental factors where IBD is emerging, such as Latin Americans.

The current increase in inflammatory bowel disease in newly industrialized countries, such as Chile, supports the role of major lifestyle transitions, among other factors might modulate the immune system through genetic adaptation. Therefore, investigating populations, such as the Chilean population, can bring essential insights into revealing the mechanisms underlying the development of IBD. Our study was designed to specifically address the role of genetic variation rather than environmental factors. Because a correlation between the percentage of Amerindian ancestry and socioeconomic conditions has been observed in Chile (22) we included only individuals belonging to the same socioeconomic strata.

The major drawback of our study is the small sample size, which must be considered while interpreting the results. While we acknowledge that the limited sample size of our study may impact the generalizability of our results, it is important to note that we obtained information from a significant proportion of individuals with inflammatory bowel disease (IBD) within the studied Metropolitan region. Although the precise prevalence of IBD in Chile is unknown, our sample size represents approximately 35% of the total number of individuals with IBD that are expected to be found in the central part of the Metropolitan region, based on estimates from the Brazilian population (16). Despite this limitation, our findings provide valuable insights into IBD's clinical and epidemiological characteristics in this population. They can motivate future research efforts to better understand this disease in Chile and other similar regions.

Due to the small sample size, we could not analyze Crohn's disease and ulcerative colitis separately, which would have limited our statistical power. While we acknowledge that this approach may have resulted in the loss of important information regarding the discrimination between these two phenotypes, we chose to focus on identifying common risk factors for all types of IBD. By doing so, we aimed to provide a comprehensive understanding of the disease that could apply to the broader IBD population.

It is important to note that smoking status was not considered in our regression model analysis due to the unavailability of data from controls. Smoking status may have influenced the observed results. However, it is worth mentioning that the prevalence of smoking among individuals with IBD in our study population was 16.3% (as shown in Table 1), which is lower than the prevalence of smoking in the general Chilean population, which is 33.4% (55).

**Figure 4:**
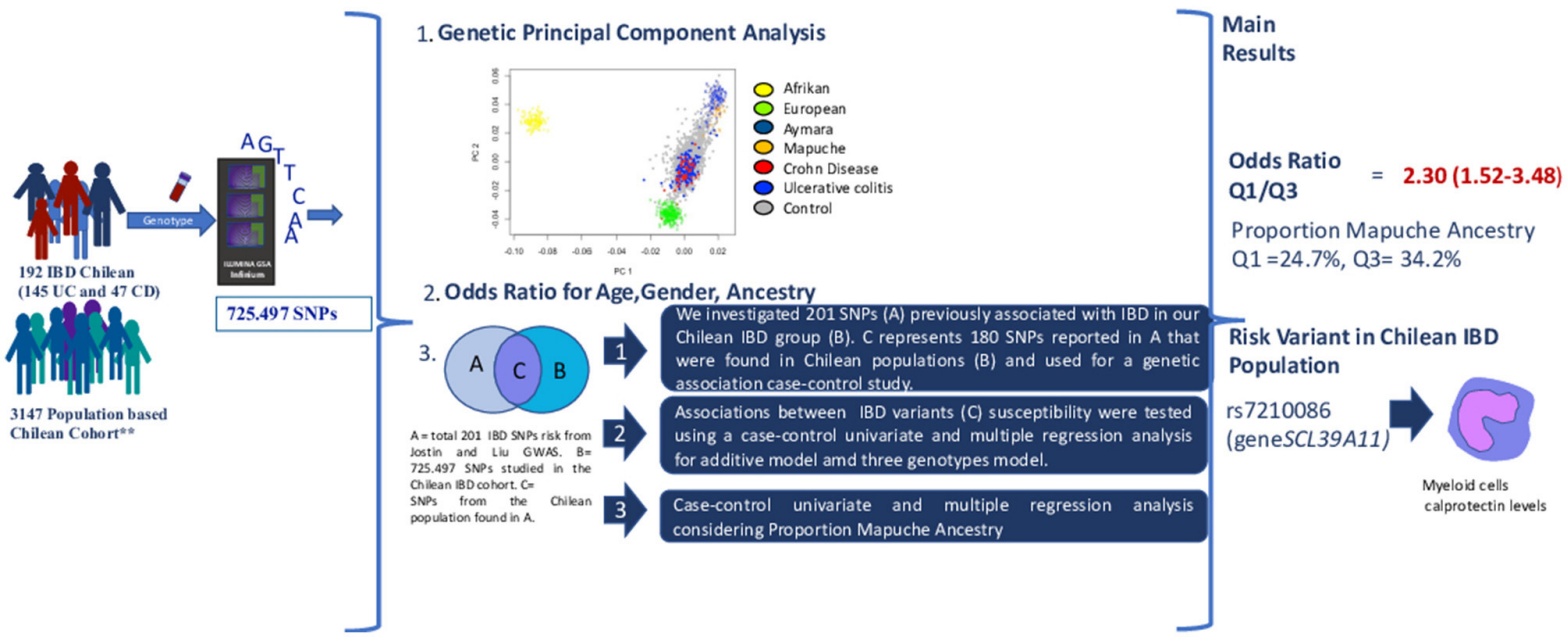
Summary workflow of the study. 192 IBD Chilean patients were genotyped for 725497 SNP using Illumina GSA Infinium. Genotype data were combined with similar information from 3,147 Chilean controls ^**^(30). (1) Proportions of Aymara, African, European, and Mapuche ancestry were estimated. (2) We calculated the odds ratios (OR) and 95% confidence intervals (CI) for gender, as well as age, and ancestry proportions. (3) We also explored associations with previously reported IBD-risk variants (8, 9) independently and in conjunction with genetic ancestry. Main results. The first and third quartiles of the proportion of Mapuche ancestry in IBD patients were 24.7 and 34.2%, respectively, and the corresponding OR was 2.30 (95% CI 1.52–3.48) for the lowest vs. the highest group. The risk variant rs7210086 related to myeloid cells was associated with IBD in the Chilean cohort (rs7210086-C, risk allele). IBD, Inflammatory bowel disease; SNPs, Single nucleotide polymorphism; GWAS, Genome wide analysis sequence; Q1, Quartile 1; Q2, Quartile 2.

However, based on our design, our results suggest that a higher PMA is associated with IBD risk. In contrast, higher PEA is associated with a lower IBD risk in our Chilean cohort. Only 1 of the 200 variants reported in Caucasians was associated with IBD risk in our population. Then, GWAS, which includes our population, is needed to identify risk variants specific to Mapuche ancestry. Interestingly, the rs7210086 variant associated with IBD is related to innate immune responses. Our workflow and main results are presented in Figure 4.

Our results encourage expanding the characterization of the immune response to an enormous array of the population- especially neglected human groups historically exposed to different environmental factors to fully understand the contribution of genetic, epigenetic, and environmental factors to immune response variants in humans.

## Data availability statement

The datasets presented in this article are not readily available because of ethical reasons. Requests to access the datasets should be directed to the corresponding authors.

## Ethics statement

Ethics approval was obtained from the Institutional Review Boards of the Pontificia Universidad Católica de Chile (IRB: 220228001) and the Servicio de Salud Metropolitano Central/HSBA (IRB: 43/2022). All the individuals provided written informed consent. The authors are not authorized by the Ethics Committee to share the raw data through public repositories. However, it is important to highlight that data sharing remains possible through academic collaboration. Researchers who are interested in accessing the data can contact the corresponding authors directly to explore potential collaboration and request access to the data. The studies were conducted in accordance with the local legislation and institutional requirements. The participants provided their written informed consent to participate in this study.

## Author contributions

TP-J: Conceptualization, Data curation, Formal analysis, Funding acquisition, Investigation, Methodology, Project administration, Resources, Supervision, Validation, Visualization, Writing—original draft, Writing—review and editing. FM: Conceptualization, Supervision, Writing—review and editing. GA: Conceptualization, Data curation, Formal analysis, Investigation, Supervision, Visualization, Writing—review and editing. DA: Data curation, Formal analysis, Supervision, Validation, Writing—review and editing. MO: Data curation, Formal analysis, Methodology, Writing—review and editing. MA-L: Conceptualization, Investigation, Writing—review and editing. CH-R: Conceptualization, Investigation, Writing—review and editing. LA: Data curation, Project administration, Writing—review and editing. NA: Data curation, Project administration, Writing—review and editing. AE: Data curation, Writing—review and editing. RE: Data curation, Writing—review and editing. SE: Data curation, Writing—review and editing. AZ: Conceptualization, Writing—review and editing. PB: Formal analysis, Writing—review and editing. VS: Data curation, Writing—review and editing. AD: Data curation, Writing—review and editing. EA: Data curation, Writing—review and editing. CP-O: Writing—review and editing. AD-A: Writing—review and editing, Data curation. DT: Formal analysis, Methodology, Supervision, Writing—review and editing. JM: Conceptualization, Data curation, Writing—review and editing. EV: Conceptualization, Writing—review and editing. MK: Supervision, Writing—review and editing. MB: Conceptualization, Formal analysis, Funding acquisition, Investigation, Methodology, Supervision, Validation, Writing—original draft, Writing—review and editing.

## References

[1] Graham DB Xavier RJ. Pathway paradigms revealed from the genetics of inflammatory bowel disease. Nature. (2020) 578:527–39. 10.1038/s41586-020-2025-2 32103191 PMC7871366

[2] Windsor JW Kaplan GG. Evolving epidemiology of IBD. Curr Gastroenterol Rep. (2019) 21:40. 10.1007/s11894-019-0705-6 31338613

[3] Agrawal M Jess T. Implications of the changing epidemiology of inflammatory bowel disease in a changing world. Unit Eur Gastroenterol J. (2022) 10:1113–20. 10.1002/ueg2.12317 PMC9752308 36251359

[4] Kaplan GG Windsor JW. The four epidemiological stages in the global evolution of inflammatory bowel disease. Nat Rev Gastroenterol Hepatol. (2021) 18:56–66. 10.1038/s41575-020-00360-x 33033392 PMC7542092

[5] Ananthakrishnan AN Bernstein CN Iliopoulos D. Environmental triggers in IBD: a review of progress and evidence. Nat Rev Gastroenterol Hepatol. (2018) 15:39–49. 10.1038/nrgastro.2017.136 29018271

[6] Peters A Nawrot TS Baccarelli AA. Hallmarks of environmental insults. Cell. (2021) 184:1455–68. 10.1016/j.cell.2021.01.043 33657411 PMC9396710

[7] Yang Y Musco H Simpson-Yap S Zhu Z Wang Y Lin X. Investigating the shared genetic architecture between multiple sclerosis and inflammatory bowel diseases. Nat Commun. (2021) 12:1–12. 10.1038/s41467-021-25768-0 34561436 PMC8463615

[8] Jostins L Ripke S Weersma RK. Host-microbe interactions have shaped the genetic architecture of inflammatory bowel disease. Nature. (2012) 491:119–24. 10.1038/nature11582 23128233 PMC3491803

[9] Liu J van Sommeren S Huang H. Association analyses identify 38 susceptibility loci for inflammatory bowel disease and highlight shared genetic risk across populations. Nat Genet. (2015) 47:979–86. 10.1038/ng.3359 26192919 PMC4881818

[10] Franke A. Inflammatory bowel disease: a global disease that needs a broader ensemble of populations. Gastroenterology. (2017) 152:14–6. 10.1053/j.gastro.2016.11.026 27888668

[11] Yang S-K Hong M Choi H Zhao W Jung Y Haritunians T. Immunochip analysis identification of 6 additional susceptibility loci for Crohn's disease in Koreans. Inflamm Bowel Dis. (2015) 21:1–7. 10.1097/MIB.0000000000000268 25489960 PMC4331109

[12] Fuyuno Y Yamazaki K Takahashi A Esaki M Kawaguchi T Takazoe M. Genetic characteristics of inflammatory bowel disease in a Japanese population. J Gastroenterol. (2016) 51:672–81. 10.1007/s00535-015-1135-3 26511940

[13] Juyal G Negi S. Genome-wide association scan in north Indians reveals three novel HLA-independent risk loci for ulcerative colitis. Gut. (2015) 64:571–9. 10.1136/gutjnl-2013-306625 24837172

[14] Wade P. Race and Ethnicity in Latin America. Lodnodn: Pluto Press (2010).

[15] Kotze PG Underwood FE Damião AOMC Ferraz JGP Saad-Hossne R Toro M. Progression of inflammatory bowel diseases throughout latin America and the Caribbean: a systematic review. Clin Gastroenterol Hepatol. (2020) 18:304–12. 10.1016/j.cgh.2019.06.030 31252191

[16] Lima Martins A Volpato RA Zago-Gomes MDP. The prevalence and phenotype in Brazilian patients with inflammatory bowel disease. BMC Gastroenterol. (2018) 18:87. 10.1186/s12876-018-0822-y 29914399 PMC6006948

[17] Klimentidis YC Miller GF Shriver MD. Genetic admixture, self-reported ethnicity, self-estimated admixture, and skin pigmentation among Hispanics and Native Americans. Am J Phys Anthr. (2009) 138:375–83. 10.1002/ajpa.20945 18951390

[18] Homburger JR Moreno-Estrada A Gignoux CR. genomic insights into the ancestry and demographic history of South America. PLoS Genet. (2015) 11:1–26. 10.1371/journal.pgen.1005602 26636962 PMC4670080

[19] NIH. Grantome. (2023). Available online at: https://grantome.com/grant/NIH/R01-DK104844-01A1 (accessed March 30, 2023).

[20] Ruiz-Linares A Adhikari K Acuña-Alonzo V Quinto-Sanchez M Jaramillo C Arias W. Admixture in Latin America: geographic structure, phenotypic diversity and self-perception of ancestry based on 7,342 individuals. PLoS Genet. (2014) 10:572. 10.1371/journal.pgen.1004572 25254375 PMC4177621

[21] Eyheramendy S Martinez FI Manevy F Vial C Repetto GM. Genetic structure characterization of Chileans reflects historical immigration patterns. Nat Commun. (2015) 6:1–14. 10.1038/ncomms7472 25778948 PMC4382693

[22] Verdugo RA Di Genova A Herrera L. Development of a small panel of SNPs to infer ancestry in Chileans that distinguishes Aymara and Mapuche components. Biol Res. (2020) 53:1–11. 10.1186/s40659-020-00284-5 32299502 PMC7161194

[23] Fuentes M Pulgar I Gallo C. Geografía génica de Chile. Distribución regional de los aportes genéticos Americanos, Europeos y Africanos. Rev Med Chil. (2014) 142:281–9. 10.4067/S0034-98872014000300001 25052264

[24] Rubin DT Ananthakrishnan AN Siegel CA Sauer BG Long MD. Clinical guideline: ulcerative colitis in adults. Am J Gastroenterol. (2019) 114:384–413. 10.14309/ajg.0000000000000152 30840605

[25] Lichtenstein GR Loftus E V Isaacs KL Regueiro MD Gerson LB Sands BE. Clinical guideline: management of crohn's disease in adults. Am J Gastroenterol. (2018) 113:481–517. 10.1038/ajg.2018.27 29610508

[26] Magro F Doherty G Peyrin-Biroulet L. ECCO position paper: Harmonization of the approach to ulcerative colitis histopathology. J Crohn's Colitis. (2020) 14:1503–11. 10.1093/ecco-jcc/jjaa110 32504534

[27] Maaser C Sturm A Vavricka SR. ECCO-ESGAR guideline for diagnostic assessment in IBD part 1: initial diagnosis, monitoring of known IBD, detection of complications. J Crohns Colitis. (2019) 13:144–64. 10.1093/ecco-jcc/jjy113 30137275

[28] Barozet E Valenzuela CY Cifuentes L. The Chilean socio-ethno-genomic cline. Biodemography Soc Biol. (2021) 66:156–71. 10.1080/19485565.2021.1879626 34182852

[29] Asociación de Investigadores de Mercado y Opinión Pública. GRUPOS Socioeconómicos de Chile. (2023). Available online at: https://aimchile.cl/gse-chile/ (accessed February 4, 2023).

[30] Barahona Ponce C Scherer D Brinster R. Gallstones, body mass index, c-reactive protein, and gallbladder cancer: mendelian randomization analysis of chilean and european genotype data. Hepatology. (2021) 73:1783–96. 10.1002/hep.31537 32893372

[31] Alexander DH Lange K. Enhancements to the ADMIXTURE algorithm for individual ancestry estimation. BMC Bioinf. (2011) 12:246. 10.1186/1471-2105-12-246 21682921 PMC3146885

[32] Genomes Project C Auton A Brooks LD. A global reference for human genetic variation. Nature. (2015) 526:68–74. 10.1038/nature15393 26432245 PMC4750478

[33] Lorenzo Bermejo J Boekstegers F González Silos R. Subtypes of Native American ancestry and leading causes of death: Mapuche ancestry-specific associations with gallbladder cancer risk in Chile. PLoS Genet. (2017) 13:1–23. 10.1371/journal.pgen.1006756 28542165 PMC5444600

[34] Reich D Patterson N Campbell D. Reconstructing Native American population history. Nature. (2012) 488:370–4. 10.1038/nature11258 22801491 PMC3615710

[35] Lindo J Haas R Hofman C. The genetic prehistory of the Andean highlands 7000 years BP though European contact. Sci Adv. (2018) 4:aau4921. 10.1126/sciadv.aau4921 30417096 PMC6224175

[36] Price AL Patterson NJ Plenge RM Weinblatt ME Shadick NA Reich D. Principal components analysis corrects for stratification in genome-wide association studies. Nat Genet. (2006) 38:904–9. 10.1038/ng1847 16862161

[37] Nimmons D Limdi JK. Elderly patients and inflammatory bowel disease. World J Gastrointest Pharmacol Ther. (2016) 7:51–65. 10.4292/wjgpt.v7.i1.51 26855812 PMC4734955

[38] Garza-González E Pérez-Pérez GI Mendoza-Ibarra SI Flores-Gutiérrez JP B-PF. Genetic risk factors for inflammatory bowel disease in a North-eastern Mexican population. Int J Immunogenet. (2010) 37:355–9. 10.1111/j.1744-313X.2010.00932.x 20518842

[39] Steiner A Reygaerts T Pontillo A. Recessive NLRC4-autoinflammatory disease reveals an ulcerative colitis locus. J Clin Immunol. (2022) 42:325–35. 10.1007/s10875-021-01175-4 34783940 PMC8821057

[40] Figueroa C Peralta A Herrera L. NOD2/CARD15 and Toll-like 4 receptor gene polymorphism in Chilean patients with inflammatory bowel disease. Eur Cytokine Netw. (2006) 17:125–30. 16840031

[41] Abecasis GR. GAS Power Calculator. University of Michigan School of Public Health. Available online at: https://csg.sph.umich.edu/abecasis/cats/gas_power_calculator/ (accesed october 13, 2023).

[42] Ananthakrishnan AN. IBD risk prediction using multi-ethnic polygenic risk scores. Nat Rev Gastroenterol Hepatol. (2021) 18:217–8. 10.1038/s41575-021-00425-5 33547451

[43] Brant SR Okou DT Simpson CL. Genome-wide association study identifies African-specific susceptibility loci in African Americans with inflammatory bowel disease. Gastroenterology. (2017) 152:206–17. 10.1053/j.gastro.2017.02.041 27693347 PMC5164948

[44] GWAS Catalog. (2023). Available online at: https://www.ebi.ac.uk/gwas/variants/rs7210086 (accessed February 4, 2023).

[45] Bush WS Moore JH. Chapter 11: genome-wide association studies. PLoS Comput Biol. (2012) 8:12. 10.1371/journal.pcbi.1002822 23300413 PMC3531285

[46] Ellinghaus D Jostins L Spain SL. Analysis of five chronic inflammatory diseases identifies 27 new associations and highlights disease-specific patterns at shared loci. Nat Genet. (2016) 48:510–8. 10.1038/ng.3528 26974007 PMC4848113

[47] Hamon R Homan CC Tran HB. Zinc and zinc transporters in macrophages and their roles in efferocytosis in COPD. PLoS ONE. (2014) 9:e110056. 10.1371/journal.pone.0110056 25350745 PMC4211649

[48] Wang S Song R Wang Z Jing Z Wang S Ma J. S100A8/A9 in inflammation. Front Immunol. (2018) 9:1298. 10.3389/fimmu.2018.01298 29942307 PMC6004386

[49] Karaky M Boucher G Mola S. Prostaglandins and calprotectin are genetically and functionally linked to the inflammatory bowel diseases. PLoS Genet. (2022) 18:1–23. 10.1371/journal.pgen.1010189 36155972 PMC9536535

[50] Cousins RJ Martin AB Aydemir TB Guthrie GJ Samuelson DA Chang SM. Gastric and colonic zinc transporter ZIP11 (Slc39a11) in mice responds to dietary zinc and exhibits nuclear localization. J Nutr. (2013) 143:1882–8. 10.3945/jn.113.184457 24089422 PMC3827636

[51] Ensembl. SLC39A11. Expression Atlas. (2023). Available online at: https://www.ensembl.org/Homo_sapiens/Gene/ExpressionAtlas?db=core;g=ENSG00000133195;r=17:72645949-73092712 (accessed February 4, 2023).

[52] Nédélec Y Sanz J Baharian G. Genetic ancestry and natural selection drive population differences in immune responses to pathogens. Cell. (2016) 167:657–69. 10.1016/j.cell.2016.09.025 27768889

[53] Quach H Rotival M Pothlichet J. Genetic adaptation and neandertal admixture shaped the immune system of human populations. Cell. (2016) 167:643–56. 10.1016/j.cell.2016.09.024 27768888 PMC5075285

[54] Klunk J Vilgalys TP Demeure CE. Evolution of immune genes is associated with the black death. Nature. (2022) 611:312–9. 10.1038/s41586-022-05349-x 36261521 PMC9580435

[55] Castillo-Riquelme M Bardach A Palacios A Pichón-Riviere A. Health burden and economic costs of smoking in Chile: the potential impact of increasing cigarettes prices. PLoS ONE. (2020) 15:e0237967. 10.1371/journal.pone.0237967 32857819 PMC7454964

