# Supplementary material for: Amerindian ancestry proportion as a risk factor for inflammatory bowel diseases: results from a Latin American Andean cohort
Source: Front Med (Lausanne). 2023 Oct 27;10:1258395. doi: 10.3389/fmed.2023.1258395 (PMC10642057; doi:10.3389/fmed.2023.1258395)
Supplement: Supplementary file 1 [file Table_1.docx]

| Three Genotypes Model | |  |  | Univariate | | | | | Multivariate | | | | |
| --- | --- | --- | --- | --- | --- | --- | --- | --- | --- | --- | --- | --- | --- |
| SNP | Genotype | Cases | Controls | OR | 2.5% | 97.5% | Pvalue | Pval Adjusted | OR | 2.5% | 97.5% | Pvalue | Pval Adjusted |
| rs7210086 | AA | 132 | 1745 | Reference | Reference | Reference |  |  | Reference | Reference | Reference |  |  |
|  | AC | 48 | 1206 | 0.52 | 0.37 | 0.73 |  |  | 0.52 | 0.36 | 0.73 |  |  |
|  | CC | 6 | 196 | 0.40 | 0.16 | 0.85 | 0.0001 | 1.94x10^-17^ | 0.39 | 0.15 | 0.84 | 0.0001 | 0.04 |

**Table S1. Single Nucleotide Polymorphisms associated with IBD in the studied group: Model Three Genotypes.**

SNP: single nucleotide polymorphism, OR: odds ratio, CI: confidence interval, PVal: p-value.
